# Supplementary material for: Applications of Delayed Luminescence for tomato fruit quality assessment across varied Sicilian cultivation zones
Source: PLoS One. 2023 Jun 1;18(6):e0286383. doi: 10.1371/journal.pone.0286383 (PMC10234554; doi:10.1371/journal.pone.0286383)
Supplement: S2 Fig — (PDF) [file pone.0286383.s003.pdf]

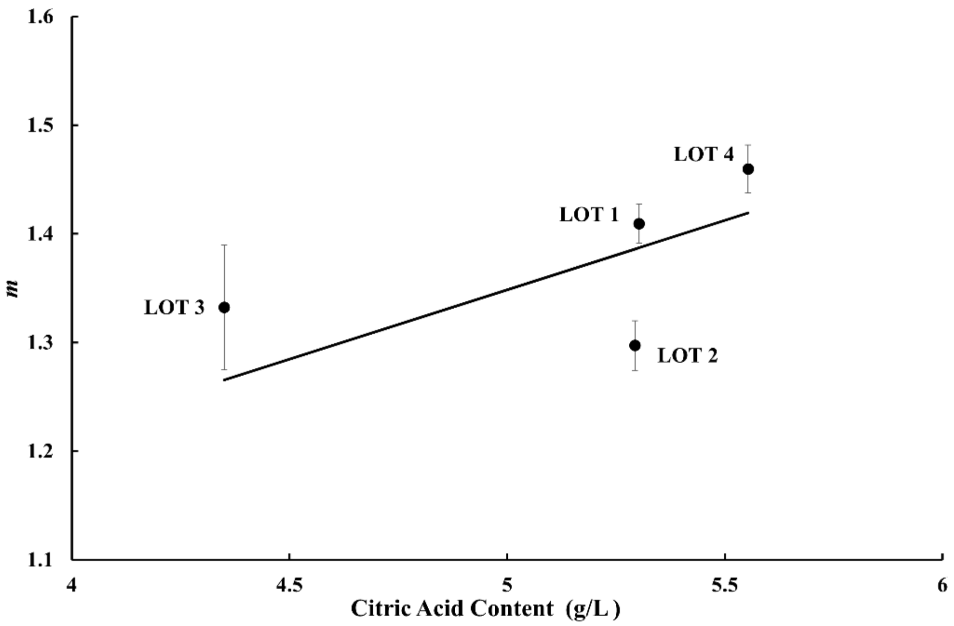

Fig S2. DL decay slope (m value) as a function of citric acid content (TA). Markers represent the average values obtained, for each Lot, from the single fruit DL trends. Bars denotes standard errors. Solid line refers to the weighted linear fit of experimental data, according to the equation $y = (0.13 \pm 0.16)\,x + (0.71 \pm 0.84)$, with $R^2 = 0.29$.
