# Supplementary material for: Applications of Delayed Luminescence for tomato fruit quality assessment across varied Sicilian cultivation zones
Source: PLoS One. 2023 Jun 1;18(6):e0286383. doi: 10.1371/journal.pone.0286383 (PMC10234554; doi:10.1371/journal.pone.0286383)

PLOS ONE

Application of Delayed Luminescence for assessing quality of tomato fruit coming from different Sicilian growing areas

--Manuscript Draft--

**Manuscript Number:**	PONE-D-23-08412
**Article Type:**	Research Article
**Full Title:**	Application of Delayed Luminescence for assessing quality of tomato fruit coming from different Sicilian growing areas
**Short Title:**	Tomato fruit quality assessed by Delayed Luminescence
**Corresponding Author:**	Salvina Panebianco, PhD<br>University of Catania Department of Agricolture Food and Environment: Universita degli Studi di Catania Dipartimento di Agricoltura Alimentazione e Ambiente<br>Catania, ITALY
**Keywords:**	Delayed Luminescence; tomato fruit; quality; ripeness; non-destructive technique
**Abstract:**	Food quality and traceability are at the center of attention by food industry, representing cornerstones in healthy eating. To best meet consumers' requests, researchers have focused on the implementation of analytical techniques able to provide information on quality parameters involved in a fast and non-destructive way. In this study, we propose to use the Delayed Luminescence, a ultra-weak and photo-induced emission of optical photons, as a tool for a rapid evaluation of quality profile associated to fruit ripening, in support of traditional analysis methods. DL measurements have been performed on cherry tomatoes, with and without the PGI certification “Pomodoro di Pachino”, harvested from two different growing areas of south-eastern Sicily. Then, DL emissions were correlated with soluble solid content and titratable acidity values, which are known to affect the flavor, the commerciality and the maturity degree of tomato fruits. In addition, we evaluated the changes in the DL parameters with respect to the geographical origin of the cherry tomatoes, with the aim of testing the possibility of applying the technique for identification purposes.<br>The signals of Delayed Luminescence appeared to be good indicators of the macromolecular structure of the biological system, revealing structural changes related to the content of total soluble solids present in the juice of the tomatoes analyzed, whereas they appeared unsuitable for authenticating vegetable crops, since the differences in the photon yields emitted by tomato Lots were not related to territory of origin. Thus, our results suggest that DL can be used as a nondestructive indicator of important parameters linked to tomato fruit quality.
**Order of Authors:**	Salvina Panebianco, PhD
	Eduard van Wijk
	Yu Yan
	Gabriella Cirvilleri
	Alberto Continella
	Giulia Modica
	Agatino Musumarra
	Maria Grazia Pellegriti
	Agata Scordino
**Additional Information:**	
**Question**	**Response**
**Financial Disclosure**<br><br>Enter a financial disclosure statement that	Funded by Incentive Plan for Research (PIA.CE.RI.) 2020-22 line 2 of the University of Catania, Research Projects “NaTI4Smart” and “MEDIT ECO”, National Operational Programme (PON) on Research and Innovation 2014-2020 “POFACS

describes the sources of funding for the work included in this submission. Review the submission guidelines for detailed requirements. View published research articles from *PLOS ONE* for specific examples.<br><br>This statement is required for submission and **will appear in the published article** if the submission is accepted. Please make sure it is accurate.<br><br>**Unfunded studies**<br>Enter: *The author(s) received no specific funding for this work.*<br><br>**Funded studies**<br>Enter a statement with the following details:<br>• Initials of the authors who received each award<br>• Grant numbers awarded to each author<br>• The full name of each funder<br>• URL of each funder website<br>• Did the sponsors or funders play any role in the study design, data collection and analysis, decision to publish, or preparation of the manuscript?<br>• **NO** - Include this sentence at the end of your statement: *The funders had no role in study design, data collection and analysis, decision to publish, or preparation of the manuscript.*<br>• **YES** - Specify the role(s) played.<br><br>* typeset	(ARS01_00640)" - Italian Ministry of University and Research. The funders had no role in study design, data collection and analysis, decision to publish, or preparation of the manuscript.
**Competing Interests**<br><br>Use the instructions below to enter a competing interest statement for this submission. On behalf of all authors, disclose any competing interests that could be perceived to bias this work—acknowledging all financial support and any other relevant financial or non-financial competing interests.<br><br>This statement is required for submission and **will appear in the published article** if the submission is accepted. Please make sure it is accurate and that any funding	The authors have declared that no competing interests exist.

sources listed in your Funding Information later in the submission form are also declared in your Financial Disclosure statement.<br><br>View published research articles from *PLOS ONE* for specific examples.<br><br>**NO authors have competing interests**<br><br>Enter: *The authors have declared that no competing interests exist.*<br><br>**Authors with competing interests**<br><br>Enter competing interest details beginning with this statement:<br><br>*I have read the journal's policy and the authors of this manuscript have the following competing interests: [insert competing interests here]*<br><br>* typeset	
**Ethics Statement**<br><br>Enter an ethics statement for this submission. This statement is required if the study involved:<br><br>• Human participants<br>• Human specimens or tissue<br>• Vertebrate animals or cephalopods<br>• Vertebrate embryos or tissues<br>• Field research<br><br>Write "N/A" if the submission does not require an ethics statement.<br><br>General guidance is provided below. Consult the submission guidelines for detailed instructions. **Make sure that all information entered here is included in the Methods section of the manuscript.**	N/A

**Format for specific study types**<br><br>**Human Subject Research (involving human participants and/or tissue)**<br>• Give the name of the institutional review board or ethics committee that approved the study<br>• Include the approval number and/or a statement indicating approval of this research<br>• Indicate the form of consent obtained (written/oral) or the reason that consent was not obtained (e.g. the data were analyzed anonymously)<br><br>**Animal Research (involving vertebrate animals, embryos or tissues)**<br>• Provide the name of the Institutional Animal Care and Use Committee (IACUC) or other relevant ethics board that reviewed the study protocol, and indicate whether they approved this research or granted a formal waiver of ethical approval<br>• Include an approval number if one was obtained<br>• If the study involved *non-human primates*, add *additional details* about animal welfare and steps taken to ameliorate suffering<br>• If anesthesia, euthanasia, or any kind of animal sacrifice is part of the study, include briefly which substances and/or methods were applied<br><br>**Field Research**<br><br>Include the following details if this study involves the collection of plant, animal, or other materials from a natural setting:<br>• Field permit number<br>• Name of the institution or relevant body that granted permission	
**Data Availability**<br><br>Authors are required to make all data underlying the findings described fully available, without restriction, and from the time of publication. PLOS allows rare exceptions to address legal and ethical concerns. See the PLOS Data Policy and FAQ for detailed information.	Yes - all data are fully available without restriction

A Data Availability Statement describing where the data can be found is required at submission. Your answers to this question constitute the Data Availability Statement and **will be published in the article**, if accepted.<br><br>**Important:** Stating 'data available on request from the author' is not sufficient. If your data are only available upon request, select 'No' for the first question and explain your exceptional situation in the text box.<br><br>Do the authors confirm that all data underlying the findings described in their manuscript are fully available without restriction?	
**Describe where the data may be found in full sentences. If you are copying our sample text, replace any instances of XXX with the appropriate details.**<br><br>• If the data are **held or will be held in a public repository**, include URLs, accession numbers or DOIs. If this information will only be available after acceptance, indicate this by ticking the box below. For example: *All XXX files are available from the XXX database (accession number(s) XXX, XXX.).*<br>• If the data are all contained **within the manuscript and/or Supporting Information files**, enter the following: *All relevant data are within the manuscript and its Supporting Information files.*<br>• If neither of these applies but you are able to provide **details of access elsewhere**, with or without limitations, please do so. For example:<br><br>*Data cannot be shared publicly because of [XXX]. Data are available from the XXX Institutional Data Access / Ethics Committee (contact via XXX) for researchers who meet the criteria for access to confidential data.*<br><br>*The data underlying the results presented in the study are available from (include the name of the third party*	All relevant data are within the manuscript and its Supporting Information files.

and contact information or URL).<br>• This text is appropriate if the data are owned by a third party and authors do not have permission to share the data.<br><br>* typeset	
Additional data availability information:	

Powered by Editorial Manager® and ProduXion Manager® from Aries Systems Corporation

Manuscript Click here to access/download;Manuscript;Manuscript.docx

Applications of Delayed Luminescence for assessing quality of tomato fruit coming from different Sicilian growing areas

Salvina Panebianco[1*], Eduard van Wijk[2], Yu Yan[2], Gabriella Cirvilleri[1], Alberto Continella[1], Giulia Modica[1], Agatino Musumarra[3,4], Maria G. Pellegriti[4], Agata Scordino[3,5]

[1]Dipartimento di Agricoltura, Alimentazione e Ambiente, Università di Catania, Catania, Italy
[2]Meluna Research, Department of Biophotonics, Wageningen, Netherlands
[3]Dipartimento di Fisica e Astronomia, Università di Catania, Catania, Italy
[4]Istituto Nazionale di Fisica Nucleare – Sezione di Catania, Catania, Italy
[5]Istituto Nazionale di Fisica Nucleare – Laboratori Nazionali del Sud, Catania, Italy

*Corresponding author
E-mail: salvina.panebianco@phd.unict.it

Abstract

Food quality and traceability are at the center of attention by food industry, representing cornerstones in healthy eating. To best meet consumers' requests, researchers have focused on the implementation of analytical techniques able to provide information on quality parameters involved in a fast and non-destructive way.

In this study, we propose to use the Delayed Luminescence, a ultra-weak and photo-induced emission of optical photons, as a tool for a rapid evaluation of quality profile associated to fruit ripening, in support of traditional analysis methods. DL measurements have been performed on cherry tomatoes, with and without the PGI certification "Pomodoro di Pachino", harvested from two different growing areas of south-eastern Sicily. Then, DL emissions were correlated with soluble solid content and titratable acidity values, which are known to affect the flavor, the commerciality and the maturity degree of tomato fruits. In addition, we evaluated the changes in the DL parameters with respect to the geographical origin of the cherry tomatoes, with the aim of testing the possibility of applying the technique for identification purposes.

The signals of Delayed Luminescence appeared to be good indicators of the macromolecular structure of the biological system, revealing structural changes related to the content of total soluble solids present in the juice of the tomatoes analyzed, whereas they appeared unsuitable for authenticating vegetable crops, since the differences in the photon yields emitted by tomato Lots were not related to

territory of origin. Thus, our results suggest that DL can be used as a nondestructive indicator of important parameters linked to tomato fruit quality.

1. Introduction

Consumers' request for high-quality foods with a certified geographical identity, like PDO (Protected Designation of Origin) and PGI (Protected Geographical Indication) products, has greatly increased in the last years, widening the fraud phenomenon due to the higher economic value of these products. As a result, analytical methods able to determine ~~quick and easy~~ the quality and the geographical origin of products become increasingly necessary. In this context, the focus of this research ~~activity~~ was to authenticate and determine the quality of ~~the~~ PGI "Pomodoro di Pachino" using non-destructive physical analysis methodologies. Results obtained by the XRF technique [1, 2] clearly showed that fruits have ~~a characterizing~~ elemental composition, correlated to their territory of origin, which allows to identify typical elements or pollutants even when they appear in trace amounts. This clearly establishes a plethora of applications aimed at preventing food fraud and ~~at~~ certifying food quality. ~~Moreover,~~ these results boost the implementation of further non-destructive physical techniques.

Several analytical techniques, such as NIRs/FIR, Raman or Nuclear Magnetic Resonance (NMR) spectroscopy, are ~~nowadays~~ widely applied in the agri-food industry to determine the degree of fruit ripeness, ~~the~~ nutritional quality of food and/or the presence of chemical residues that are dangerous to human health [3-8]. Some of these techniques have also been successfully used for detecting ~~the~~ geographical origin of different vegetal crops~~, including the Sicilian PGI 'Pomodoro di Pachino'~~ [9, 10]. Among the non-destructive physical techniques, Delayed Luminescence (DL) has also attracted particular interest in the last decades. It measures the optical photons released by a sample as a result of its excitation by light, long time after the switching off of the illumination source. The phenomenon of delayed light emission from biological systems (in a ~~period of~~ few seconds) following exposure to illumination was ~~for the first time~~ observed in green plants in 1951 [11]. ~~In this case,~~ the amount of light emitted ~~is something like~~ 10^3-10^5 times lower in intensity with respect to fluorescence, a low-level signal whose lifetime spectrum spans from 10^{-7} to more than 10 s. This phenomenon has been associated to triplet- or metastable-state species exhibiting intrinsically long lifetimes.

Nowadays, we know that DL emission is not only associated to green plants, ~~as in general~~ [illegible] an indicator of the biological state ~~of the system~~. This ~~fact~~ allowed to explore the ~~applicative~~ potential of ~~the~~ DL in different fields, from quality control to clinical ~~investigation~~. In the agri-food sector, the technique has been used to assess the germination capacity of seeds [12, 13], ~~the~~ photosynthetic

activity in plant leaves and the ripening of fruits that lose chlorophyll as a result of their ripening [3]. In this regard, a study carried out on tomato fruits at different stages of ripeness has highlighted significant changes in the emissions of DL related to the color of fruits and to their respiration [14]. In the biological systems, DL phenomenon has been correlated to the dimension of the ordered structures where it has been measured, exhibiting drastic changes when the structure is altered. A model that assumes that DL is connected with the formation and dissociation of non-linear coherent self-trapped (localized) electron states (solitons and electro-solitons) has been implemented and it succeeded in explaining a certain number of DL features [15-18]. For this reason, it is reasonable to infer that fruits grown in different farmlands, thus having a different chemical-physical composition, can give rise to different light signals, which can be used to draw information about quality parameters.

In fruit and vegetable products, quality depends on several physical-chemical parameters (skin color, sugar and organic acid content, pH, moisture, ash content, etc.) that are not always easily detectable. These factors are closely related to the degree of ripeness of the fruit and are often used to determine the best period for harvesting. Fruit ripening is a complex process that involves irreversible changes in color, texture, taste and chemical composition. At present, the appropriate time for harvesting is mainly estimated by counting of days after flowering or through color and texture visual inspections (subjective parameters), while the chemical and physical parameters, linked to quality, are measured through destructive techniques, by using traditional instruments, like refractometer and pH meter, that require large number of samples to represent the variability within the field.

In this context, the main objective of this study was to investigate the possibility of using DL technique as alternative to traditional analysis methods in order to determine in a fast and non-destructive way the quality and/or degree of fruit ripeness. We analyzed the correlations between the DL parameters (obtained by the DL decay curves) and the physical-chemical parameters associated to fruit ripening (color, TSS, titratable acidity), carrying out simultaneous DL, chemical and colorimetric measurements on a large number of fruits, representative of 4 different tomato Lots collected from two different agricultural areas of south-eastern Sicily. In detail, two Lots came from greenhouses located in Ispica, within the Pachino district (PGI-site), and the other two from greenhouses in the province of Ragusa (non-PGI sites). When comparing Lots from different farms, we also considered the possibility of authenticating the PGI Pachino tomato by using DL technique, evaluating possible correlations between DL parameters and the geographical origin of the samples.

2. Materials and methods

2.1 Fruit sampling

Cherry tomato fruits cv. Creativo were harvested in four different greenhouses located in south-eastern Sicily, from PGI and non-PGI sites, at the same time. In detail, the area of origin of the tomato Lots included two greenhouses located in Ispica municipality (PGI sites), within the Pachino district, (Lots 3 and 4) and two greenhouses located in Marina di Ragusa and Santa Croce Camerina municipalities (non-PGI sites), in the province of Ragusa (Lots 1 and 2). A total of four Lots were collected, taking one Lot from each greenhouse (Fig 1).

In Ispica, the tomato cultivation was carried out following the rules indicated in the Pachino tomato production specifications, providing irrigation by groundwater from local wells, characterized by salty water because of the nearby sea (electrical conductivity ranging from 1500 to 10000 µS/cm). The salinity of the irrigation water in Marina di Ragusa and Santa Croce Camerina is generally lower than in Ispica.

Considering the large size of the greenhouses, for each sampling, tomatoes were randomly harvested from different points and pooled in a single Lot. In this way, it was possible to obtain a uniform and representative Lot from each greenhouse. Tomato fruits were aseptically removed from plants together with their bunches, put in plastic bags and transported to the laboratory.

In the framework of this research activity, we have analyzed tomato samples at full ripeness stage (skin with intense red color). For each Lot, 35 ripe fruits were selected, for a total of 140 fruits constituting the whole experimental set. The selected fruits appeared similar to each other in color, size and weight (Fig 2). Part of these fruits were employed to assess the physic-chemical parameters concerning tomato quality, while the rest were subjected to DL measurements, as specified below. After harvesting, and throughout the period of measurements, tomatoes were kept at room temperature and in dark conditions.

Fig 1. Area of origin of tomato Lots collected for this study. The Lots 3 and 4 were certified with the label PGI. The map was created using the program GPS Visualizer (GPS Visualizer: Leaflet Maps output).

Fig 2. Cherry tomato fruits selected for DL measurements. The Lots 1 and 2 were collected from non-PGI sites, whereas the Lots 3 and 4 from farms in the Pachino district, recognized by the European Community with the label PGI (see Fig 1).

2.2 Physical-chemical parameters

For each Lot, the size and weight measurements have been performed on 20 tomato fruits, the same fruits used for DL measurements. Fruit weights were determined using a digital balance (Diamond,

model 500 with an accuracy of 0.1 g). Fruit sizes (equatorial diameters) were measured with an electronic digital caliper (Digital Caliper 150 mm, accuracy of 0.01 mm); this measurement was repeated twice.

Due to the destructive ~~character~~ of chemical measurements, a different set of 15 fruits were selected for each Lot, ~~by using these fruits also~~ for chromatic measurements. The chromatic data ~~were~~ obtained by using a portable colorimeter model PCE-XXM 30 (PCE-Deutschland GmbH, Meschede, Germany) with a repeatability of $\Delta E^*_{ab} \leq 0.1$. A lamp with a wavelength of 400 - 700 nm (LED lamp) ~~is~~ built into the colorimeter as a light source. The measuring spot of the colorimeter ~~is~~ 8 mm in size. Fruit chromaticity was recorded according to the L^* a^* and b^* color space coordinates defined by the International Commission on Illumination (CIELAB). For each Lot, L^* a^* and b^* chromatic values were determined on the 15 fruits, ~~carrying out the~~ measurements at two different points on each fruit (for a total of 30 measures per Lot).

The L^* value determines the amount of light reflected. The a^* value indicates the chromaticity on the red (+ values) or green (− values) axis, and the b^* value indicates the chromaticity of the yellow (+ values) or blue (− values) axis [19].

From ~~the~~ a^* and b^* values we ~~have obtained the~~ a^*/b^* color ratio, ~~the~~ chroma (C^*) and ~~the~~ hue angle (H°), ~~calculated~~ on the basis ~~on the following~~ equations reported by [20]:

- $chroma = \sqrt{(a^{*2} + b^{*2}}$;
- $H° = \tan^{-1}(b^*/a^*)$ (when $a^* > 0$ and $b^* \geq 0$) or
- $H° = 180 + \tan^{-1}(b^*/a^*)$ (when $a^* < 0$).

~~As concerning~~ the chemical measurements, the fruits were crushed in order to determine ~~the values of~~ pH, total soluble solid (TSS) content, expressed as °Brix, and titratable acidity (TA) expressed as g/L of citric acid equivalent. Maturity index (MI) was calculated as the ratio between TSS and TA. For each Lot, the juice of five fruits per three replicates was extracted using a commercial juice extractor. The TSS content was determined ~~by~~ using a digital refractometer (Atago CO., LTD, model PR-32 α, Tokyo, Japan) with a °Brix detection range of 0% to 32%, whereas pH and TA of the tomato juices were determined using an automatic titration device (Hach, TitraLab AT1000 Series, Lainate, Milano) with 0.1 N NaOH up to pH 8.1.

All experimental data were analyzed through the one-way analysis of variance (ANOVA StatSoft Inc, Tulsa, OK, USA) at an error level of 0.05 and compared by means of Fisher's test to establish possible significant differences among the various parameters.

2.3 DL measurements

The apparatus used for measuring the delayed light emission was provided by Meluna Research (Wageningen, The Netherlands) and described in [21, 22]. It included a black chamber with a sample holder suitable for a 10 cm Petri dish. The dark chamber was connected to a photomultiplier tube installed in a vertical position (PMT, model 9558QB; Electron Tubes Enterprises Ltd., Ruislip, UK). The PMT, enhanced for single photon counting, was sensitive in the 300-800 nm wavelength range. ~~Moreover~~, it was cooled to -25°C ~~in order~~ to reduce the PMT thermal noise. The samples were illuminated for 10 s by using a white tungsten halogen lamp with an emission spectrum ranging from 350 to 950 nm (20 W, model 284-2812, Philips, Germany). The number of counts ~~was~~ recovered by a time binning of 100 ms, during 30 s after excitation, thus resulting in DL spectra of 300 data points. The amplification of the photon emission signal was carried out by using a fast spectroscopy preamplifier (model 9301 ORTEC, Oak Ridge, TN) connected to the PMT, whereas data acquisition was performed by using a photon counting card (C9744; Hamamatsu, Iwata, Japan) installed ~~within~~ a personal computer. One measurement was performed on each fruit.

3. Results

3.1 Physical-chemical parameters

Table 1 reports size, weight and chromatic parameters as defined by the CIELAB system (L^* a^* and b^* coordinates, C* and H° measurements, a^*/b^* color ratio) obtained ~~for~~ the PGI and non-PGI tomato Lots.

Equatorial size and weight values were the same in most ~~of the~~ tomato fruits analyzed (only the Lot 1 was slightly larger than the others). These values ranged between 11.5-13.4 mm and 26.3-27.9 g, respectively.

~~As regard~~ the colorimetric measurements, worth ~~to note~~ that fruits of each Lot ~~have been~~ selected by visual inspection. To test their color uniformity, the color difference ΔE^*_{ab} (in the CIELAB color system) between fruits of the same Lot ~~was~~ evaluated. The average value and its standard deviation ~~resulted~~ 2.8 ± 0.9 for Lot 1, 2.9 ± 0.6 for Lot 2, 3.5 ± 1.0 for Lot 3 and 4.2 ± 1.0 for Lot 4. All the values ~~are quite~~ close to ~~the just~~ noticeable difference. ~~On the opposite~~, differences in color parameters were observed between the different Lots analyzed. In particular, the values of red - green (a^*) and yellow - blue (b^*) components, as well as the chroma and hue angle (H°) values, were slightly larger for ~~the~~ PGI certified tomatoes ~~coming~~ from Ispica (Lots 3 and 4), whereas the highest values of the a^*/b^* ratio were found for non-PGI tomato Lots ~~coming~~ from Ragusa province (Lots 1 and 2) (Table 1).

As regard the chemical parameters, the TSS content was significantly different in the analyzed tomato Lots and did not depend on the PGI quality certification of fruits (Table 2). In detail, TSS content was highest in the Lots 1 and 4. On the opposite, no significant differences in the pH, citric acid and maturity index were detected among the Lots.

Table 1. Equatorial size, weight and chromatic parameters (CIELAB system coordinates: *L, brightness; *a**, red-green component; *b**, yellow-blue component; *C**, chroma; H°, hue angle; *a*/b**, color ratio) evaluated in tomato fruits coming from two different Sicilian growing areas.**

	Lot 1	Lot 2	Lot 3	Lot 4
**Equatorial size (mm)**	27.9 ± 1.39 a	26.7 ± 1.31 b	26.9 ± 1.92 b	26.3 ± 1.49 b
**Weight (g)**	13.4 ± 1.6 a	11.5 ± 1.4 b	11.7 ± 2.2 b	11.7 ± 1.7 b
L*	32.4 ± 1.01 c	33.3 ± 0.85 b	34.0 ± 1.45 a	33.9 ± 1.53 ab
a*	12.8 ± 1.75 b	14.5 ± 1.47 a	14.2 ± 1.61 a	14.2 ± 1.85 a
b*	15.8 ± 1.08 c	17.5 ± 1.52 b	18.8 ± 1.84 a	19.0 ± 2.31 a
C*	20.4 ± 1.68 c	22.4 ± 2.19 b	21.4 ± 2.05 bc	23.6 ± 2.29 a
**H°**	51.2 ± 3.15 c	50.6 ± 2.57 c	57.9 ± 3.98 a	53.0 ± 2.21 b
a*/b*	0.81 ± 0.09 a	0.83 ± 0.07 a	0.76 ± 0.06 b	0.75 ± 0.09 b

The size values represent the mean of 40 measures per Lot ± standard deviation (two measurements per 20 fruits); weight values represent the mean of 20 measures per Lot ± standard deviation; chromatic parameters, for each Lot, represent the mean of 30 measures ± standard deviation (two measurements per 15 fruits). The Lots 1 and 2 were harvested in non-PGI sites, whereas the Lots 3 and 4 in Pachino district (PGI-sites). Values followed by different letters within each row are significantly different at $\alpha \leq 0.05$ (Fisher's least significant difference test).

Table 2. Chemical parameters evaluated in tomato fruits coming from two different Sicilian growing areas.

	Lot 1	Lot 2	Lot 3	Lot 4
**TSS (Brix°)**	8.1 ± 0.2 b	7.1 ± 0.3 c	7.2 ± 0.3 c	8.6 ± 0.3 a
**pH**	4.80 ± 0.12 a	4.57 ± 0.13 a	4.83 ± 0.09 a	4.57 ± 0.20 a
**Citric acid (g/L)**	5.30 ± 0.22 a	5.29 ± 0.74 a	4.35 ± 1.32 a	5.55 ± 0.14 a
**Maturity index (TSS/TA)**	1.52 ± 0.08 a	1.35 ± 0.21 a	1.74 ± 0.45 a	1.55 ± 0.03 a

For each Lot, values represent the mean of 3 measures ± standard deviation. For each Lot, measurements were carried out on 3 replicates consisting of the juice of 5 tomato fruit). The Lots 1 and 2 were harvested in non-PGI sites, whereas the Lots 3 and 4 in Pachino district (PGI-sites). Values followed by different letters within each row are significantly different at $\alpha = 0.05$ (Fisher's least significant difference test).

3.2 DL measurements

We have measured the ultraweak delayed photon emission (Delayed Luminescence, DL) by tomato fruits following light irradiation, by analyzing cherry tomato fruits belonging to four different Lots, one for each farm described in par. 2.1. In particular, for each Lot, DL measurements have been

performed on 20 fruits (for a total of 80 measurements), characterized by similar color, size and weight.

The photon count rate, recorded over time after the light was turned off, was plotted to evaluate the trend of the decay curves. The decay curves recorded for each Lot were superimposed in order to study the internal variability of the four lots. The DL decay curves obtained for the 20 tomato fruits belonging to the four tomato Lots are shown in Fig 3.

Excluding ~~the~~ Lot 3, ~~the~~ total DL photon yield emitted by tomato fruits ranged within one order of magnitude. The decay trends of Lots 1, 2 and 4 were found quite homogeneous, ~~being~~ obtained by tomatoes with similar features. ~~The variability within the Lot 3 was instead significantly higher than that observed for the other Lots, even if there is no evidence of differences, as for instance in color, between fruits of this Lot with respect the other Lots.~~

By comparing the decay curves obtained from tomatoes belonging to different Lots, it was possible to unveil differences in the emission rate. In detail, the intensity of ~~the~~ DL signals at t = 0.1s (first point of the decay curve) was larger for ~~the~~ Lots 1 and 4 and lower for ~~the~~ Lots 2 and 3, suggesting that differences between Lots ~~can~~ be related to their overall composition/structure rather than their geographical origin.

~~In order to~~ quantify the differences in ~~the~~ trends of delayed light emitted by ~~the~~ different Lots, thus simplifying their comparisons, experimental data were fitted ~~by~~ using the following empirical decay function, ~~firstly~~ introduced by E. Becquerel in 1867:

$$I(t) = \frac{I_0}{\left(1 + \frac{t}{t_0}\right)^m} \qquad (1)$$

This function has been largely used to describe DL decays obtained by a great variety of biological systems [23, 24] and it has already been used to characterize relaxation of other complex systems [25-28]. Indeed, relaxation of complex systems from non-equilibrium state towards equilibrium cannot be characterized by single rate coefficient but more appropriately a distribution of relaxation kinetics exists, which in turn can be related to the hierarchically organized structure of the energy landscape [25].

Fit parameters were evaluated according to the non-linear least-squares procedure described in [23]. Figure 4 shows the parameters m and t_0 of Eq (1) obtained after normalization of data to the first experimental point, i.e. the photon rate I_1 at t = 0.1s. The I_1 values as a function of the fit parameter m ~~is~~ reported in Fig 5.

In addition, we have developed a fast fitting procedure ~~in order~~ to obtain ~~the~~ DL parameters in the shortest possible time. For this purpose, we tried to obtain a single representative trend for each Lot by using all the normalized measurements of the Lot, and evaluating, at each time instant, the mean value of the corresponding 20 DL rates at that instant, ~~so~~ getting an average normalized decay trend. Figure 6 reports the average normalized decay trends obtained for the four Lots, as ~~above~~ explained, along with the corresponding theoretical trend Eq (1) (solid line). The parameters t_0 and m obtained through the fitting procedure applied on the averaged decay curves ~~are~~ reported in Fig 7 and compared with the Lot' mean value of the DL fit parameters obtained averaging on the full set of fruit data. It appears that percentage differences between the mean value and ~~the~~ corresponding value of the averaged trend ~~are~~ less than 5% for all the Lots, except for Lot 3. These results suggest that fitting of average normalized DL decay curves ~~is~~ a valuable tool to use when a rapid analysis of large amounts of data is required.

The parameters t_0 and m obtained through the fitting procedure of decay curves associated to PGI and non-PGI tomato Lots were also compared with each other in order to verify the possibility of using luminescence for identification purposes.

By comparing data reported in Figs 4, 5 and 7, it was possible to highlight significant differences between the Lots analyzed, especially in the slopes (parameter m) of the hyperbolic trends. ~~As it regards~~ the mean values of DL parameters obtained from decay curves of 20 fruits (see Fig 7, white bars), the maximum values for the slopes were found for ~~the~~ Lots 1 and 4. Significant differences were also found for the parameter t_0 and ~~the~~ experimental first point I_1. As previously observed for TSS measurements, these differences did not depend on the geographical origin of the Lots.

Fig 3. DL decay curves of single fruits per Lot. DL measurements have been performed on 20 fruits per Lot, identified with different colors**.** Data are plotted on a log-log scale.

Fig 4. Comparison between fit parameters. DL parameters m and t_0 obtained through the fitting procedure according to Eq. (1) of the DL normalised decay curves in correspondence of each fruit which was part of a Lot: (diamond) Lot 1, (square) Lot 2, (circle) Lot 3, (triangle) Lot 4. Errors are within the marker size. Full markers represent mean values ±. S.E. for each Lot.

Fig 5. Comparison between experimental data and decay slope. First experimental point I_1 of DL decay as a function of the parameter m obtained through the fitting procedure according to Eq (1) of the DL normalised decay curves in correspondence of each fruit which was part of a Lot: (diamond) Lot 1, (square) Lot 2, (circle) Lot 3, (triangle) Lot 4. Errors of m parameter are within the marker size. Poissonian errors (square roots) of the count I_1 are omitted for the sake of clarity. Full markers represent mean values ±. S.E. for each Lot.

Fig 6. Averaged normalized decay trend. For each tomato Lot under study, the average normalized decay trend was evaluated (see text). Solid lines refer to the theoretical trend according to Eq. (1). Data are plotted on a log-log scale.

Fig 7. Comparison between DL parameters obtained by using two different procedures. DL parameters obtained according to Eq (1) are reported for each Lot: (white columns) mean values of the DL parameters obtained by fitting the single fruit DL decay trends, (grey columns) DL parameters obtained by fitting the averaged normalized decay trend. Bars indicate the standard errors. (a) t_0 parameter, (b) *m* parameter.

4. Discussion

Chromatic and chemical parameters determined in this study play an important role in the agri-food sector for quality management, since they are widely used to check quality standards during vegetable processing or to determine their ~~time of~~ harvesting. Among chromatic parameters, the *a*/b** color ratio defined in the CIELAB system has been proposed by [29] as suitable for establishing the degree of tomato ripeness, depending on how much its value is close to one. More recently, other authors have highlighted positive correlations between the *a*/b** ratio and the lycopene content, whose biosynthesis increases greatly during the tomato ripening process due to the conversion of chloroplasts in chromoplasts, taking the place of chlorophyll [20, 30]. In addition to the chromatic indices, ~~also~~ TSS or TA values were related to the ripening process of fruits, strongly affecting sweetness, sourness and flavor of fresh and processed horticultural products [31]. The TSS value indicates ~~the amount of~~ dissolved solids (sugars and organic acids, together with small amounts of vitamins, proteins, pigments, phenolics and minerals) present in a food [32]. Since sugars (glucose and fructose) constitute most of the total soluble solids in a tomato fruit (approximately 65-70%) [30], ~~the~~ TSS content for this category of food could be associated in good approximation with the sugar content and, consequently, with ~~the sweetness of the~~ fruit.

In this study, statistical analysis of the colorimetric data highlighted that non-PGI tomatoes (Lots 1 and 2) were redder, and ~~so~~ riper, than ~~the~~ PGI tomatoes (Lots 3 and 4), whereas chemical parameters showed similarity, especially in solid soluble (TSS) content, between Lots 1 and 4 from one side and Lots 2 and 3 on the other side. ~~So~~, sweetness cannot be associated ~~neither~~ to the geographical origin or to ~~the~~ agricultural practices distinguishing ~~the~~ PGI tomatoes from non-PGI ones~~, in this case~~.

The ~~chemical-physical changes~~ induced in biological systems by biotic and abiotic factors can be brought to light also through non-destructive physical techniques. A biological system suitably irradiated with a source of electromagnetic radiation in different spectral bands will generate in response different signals in relation to its qualitative profile, defined by the content of chemical elements and organic compounds present in it. Spectroscopic methods involving electromagnetic radiations, such as Raman, NIRs or XRF spectroscopy are sensitive to changes involving food

chemical composition ~~and, consequently, they~~ are widely used to discriminate foodstuffs on the basis of their geographical origin or cultivation techniques [33-35] or to detect the presence of chemical pesticides and toxic substances, such as mycotoxins [5, 36, 37].

In this ~~paper~~, we tested the possibility to apply DL technique as a tool to discriminate tomato Lots of the same cultivar subjected to different agronomic practices and farming conditions (PGI and non-PGI tomato samples) based on some parameters linked to the sensory quality of ripe fruits. In this ~~respect~~, the possibility of testing and quantifying differences between vegetable crops through non-destructive methods are of great industrial relevance.

The comparisons made on t_0 and *m* parameters associated to decay curves of PGI and non-PGI tomatoes highlighted significant differences between the four Lots analyzed. As previously observed for TSS measurements, the slopes of curves reached the highest values for ~~the~~ Lots 1 and 4. Similarly, ~~also~~ the values associated with the parameter t_o and ~~the~~ experimental first point I_1 differed in the four tomato Lots analyzed. Overall, it was observed that Lots from the same growing area ~~exhibit~~ different DL decay curves ~~and that, conversely~~, Lots from different growing areas had similar temporal trends. Thus, the analysis of ~~the~~ DL data set actually does not support ~~a~~ classification by production areas and, consequently, it is not suitable for authenticating vegetal foodstuffs. This should not be surprising, if we consider the kind of information that can be retrieved by this technique. ~~In fact, unlike~~ other optical spectroscopies, Delayed Luminescence is hardly applicable to quantitative compositional analyses or Lot authentication, as the counted photons cannot be directly related to characterizing chemical components. As said, DL is an indicator of the functional state of a biological system, as it highlights structural and/or functional changes in cells induced by certain metabolic processes [38, 39]. External agents that affect plant photosynthesis or chloroplast content (physiological stresses, atmospheric pollutants, agricultural practices, etc.) often induce structural changes in biological systems that in turn lead to changes in the number of optical photons released [3]. In this ~~respect~~, recent studies have shown that biological organisms and herbal medicines can release different light signals as growing environments, agronomic practices and technological processes vary [22, 40-42]. However, based on our preliminary results, we believe that it is difficult to correlate any structural changes to the territory of origin, especially in the case of ripe fruit growing under similar climatic conditions, as numerous variable can arise inducing different DL signals.

~~In order to~~ assess whether luminescence can be used as indicator of quality, ~~the~~ DL parameters were correlated with the chemical parameters previously determined for each Lot. ~~So, in order to~~ perform a correlation analysis, we ~~have~~ considered the slopes of the DL curves (*m* parameter), whose average values ~~shows up~~ the largest variability between Lots (see Fig 7b). In particular, the correlations

between the *m* parameter and TSS, TA or MI were studied by considering the mean values of the parameters on the entire set of data.

As previously reported, sugars, acids and their ratio are important to determine sweetness, sourness and overall flavor intensity [31]. Tomato fruits with a high level of acids and sugars have ~~a~~ good flavor, while those with a low level of acids or sugars result in insipid or tart tomatoes, respectively. The TSS, TA and TSS/TA parameters are also used to determine the best time for harvesting, since they change considerably during fruit ripening. ~~In fact~~, TSS content tends to increase as a result of biosynthesis of sugars, while TA increase~~s~~ during tomato development, reaching a maximum at the breaker stage, and then ~~it~~ decrease~~s~~ as ripening progresses [43].

The results, shown in ~~the~~ Fig 8, revealed linear relationship (R^2 = 0.994) between the fit slopes (*m* values) and ~~the~~ measured TSS parameters. Figure 8 also displays as a solid line the linear trend obtained by fitting data through weighted linear regression method. Interestingly, a still good relationship (R^2 = 0.976) holds if we use the *m*-parameters obtained by according to Eq (1) the averaged normalized decay trend of each lot of Fig 6. No correlation (R^2 = 0.29) or poor correlation (R^2 = 0.49) ~~can be revealed~~ between *m* and citric acid content or maturity index, respectively (data not shown).

The phenomenological connection between quality and photon emission rate (to which the *m* parameter is correlated) has been found for other samples and are reported in literature. In this regard~~s~~, some studies have found correlations between DL signals and color of tomato fruits at different maturity stages [14], as well as between DL intensity and fungal contamination level in peanuts artificially inoculated with *A. flavus* [44]. As previously reported, ~~recently,~~ DL measurements were found ~~able also~~ to differentiate medicinal herbs prepared under different conditions and showing different age and therapeutic properties (these characteristics reflect the presence of different active chemical compounds) [21, 22, 40]. ~~Consequently~~, our preliminary results are in agreement with findings reported in literature and suggest a possible use of DL technique as an indicator of ~~the~~ fruit internal quality.

Moreover, due to the negligible differences between the DL parameters as a result of the two fitting procedures, the one that consider average parameters starting from the single fruit DL decay and the other that instead consider the parameters of the averaged decay as above explained, it can be assessed that fitting of average normalized DL decay curves is a valid tool to be used for fast analysis of a large amount of data.

Fig 8. Comparison between chemical and DL features. DL decay slope (m value) as a function of the total soluble solid content (TSS) is reported. Markers represent the average values obtained, for each Lot, from the

single fruit DL trends. Bars denotes standard errors. Solid line refers to the weighted linear fit of experimental data, according to the equation y = (0.11 ± 0.01) x + (0.54 ± 0.05), with R^2 = 0.994.

5. Conclusions

The feasibility of DL technique in determining some relevant quality features affecting the flavor, the commerciality and the maturity degree of vegetal crops was investigated on cherry tomato samples harvested at full ripeness stage (fully red skin) from two different growing areas of south-eastern Sicily. In addition, we have also explored the possibility of applying the DL technique in order to disentangle tomato Lots on the basis of their geographical origins. The tomato fruits under study appeared similar in colour, pH and acid content, while differing in the total soluble solid content.

The preliminary results achieved in this study suggest that DL is not suitable for authenticating vegetal foodstuffs, as the changes in the DL parameters [illegible]ociated to the different DL decay curves (*m* and t_0 values) were not correlated to the territorial origin of the Lots. Briefly, investigating by a technique showing the functional properties of the biological sample it is difficult to discriminate against similar products that, on the other side, can differ from each other by a few characterizing chemical components.

Conversely, the differences in the photon decay rate emitted by the tomato fruits after their illumination reflected the differences in the content of total soluble solids present in their juice. Thus, we believe that the DL technique has a good potential, being a rapid technique which can help the farmers to understand, in real time, the quality of their products. The assessment of the flavor and the maturity stage of tomato fruits through the DL technique, in addition or in place of the traditional chemical methodologies, would offer in the agri-food sector many advantages, and in particular, it would allow a fast quality assessment without damaging or destroying the analyzed biological matrices, thus providing a large number of measurements in a short time and more accurate results.

References

1. Panebianco S, Mazzoleni P, Barone G, Musumarra A, Pellegriti MG, Pulvirenti A, et al. Feasibility study of tomato fruit characterization by fast XRF analysis for quality assessment and food traceability. Food Chem. 2022; 383: 132364. https://doi.org/10.1016/j.foodchem.2022.132364
2. Panebianco S, Barone G, Cirvilleri G, Finocchiaro C, Mazzoleni P, Musumarra A, et al. A feasibility study of tomato fruit quality assessment and traceability by X-ray fluorescence spectroscopy and statistical analysis. Il Nuovo Cimento C, 2022; 45(6): 1-4. 10.1393/ncc/i2022-22208-3

3. Abbott JA. Quality measurement of fruits and vegetables. Postharvest Biol. Technol. 1999; 15(3): 207-225. https://doi.org/10.1016/S0925-5214(98)00086-6
4. Liu Y, Sun X, Ouyang A. Nondestructive measurement of soluble solid content of navel orange fruit by visible–NIR spectrometric technique with PLSR and PCA-BPNN. LWT-Food Sci. Technol. 2010; 43(4): 602-607. https://doi.org/10.1016/j.lwt.2009.10.008
5. Teye E, Huang XY, Afoakwa N. Review on the potential use of near infrared spectroscopy (NIRS) for the measurement of chemical residues in food. Am. J. Food Sci. Technol. 2013; 1(1): 1-8. http://pubs.sciepub.com/ajfst/1/1/1
6. de Oliveira GA, de Castilhos F, Renard CMGC, Bureau S. Comparison of NIR and MIR spectroscopic methods for determination of individual sugars, organic acids and carotenoids in passion fruit. Food Res. Int. 2014; 60: 154-162. https://doi.org/10.1016/j.foodres.2013.10.051
7. Sobolev AP, Mannina L, Proietti N, Carradori S, Daglia M, Giusti AM, et al. Untargeted NMR-based methodology in the study of fruit metabolites. Molecules 2015; 20(3): 4088-4108. https://doi.org/10.3390/molecules20034088
8. Li JL, Sun DW, Cheng JH. Recent advances in nondestructive analytical techniques for determining the total soluble solids in fruits: a review. Compr. Rev. Food Sci. Food Saf. 2016; 15(5): 897-911. https://doi.org/10.1111/1541-4337.12217
9. Mallamace D, Corsaro C, Salvo A, Cicero N, Macaluso A, Giangrosso G, et al., A multivariate statistical analysis coming from the NMR metabolic profile of cherry tomatoes (The Sicilian Pachino case). Physica A 2014; 401: 112-117. https://doi.org/10.1016/j.physa.2013.12.054
10. Masetti O, Ciampa A, Nisini L, Sequi P, Dell'Abate MT. A multifactorial approach in characterizing geographical origin of Sicilian cherry tomatoes using ^{1}H-NMR profiling. Food Res. Int. 2017; 100: 623-630. https://doi.org/10.1016/j.foodres.2017.07.059
11. Strehler B, Arnold W. Light production by green plants. J. Gen. Physiol. 1951; 34: 809–820. doi: 10.1085/jgp.34.6.809
12. Grasso R, Abe T, Cirrone GAP, Cuttone G, Gulino M, Musumeci F, et al. Effects of ion irradiation on seedlings growth monitored by ultraweak Delayed Luminescence. PLoS One 2016; 11(12): e0167998. https://doi.org/10.1371/journal.pone.0167998
13. Grasso R, Gulino M, Giuffrida F, Agnello M, Musumeci F, Scordino A. Non-destructive evaluation of watermelon seeds germination by using Delayed Luminescence. J. Photochem. Photobiol. B. 2018; 187: 126-130. https://doi.org/10.1016/j.jphotobiol.2018.08.012
14. Triglia A, La Malfa G, Musumeci F, Leonardi C, Scordino A. Delayed luminescence as an indicator of tomato fruit quality. J. Food Sci. 1998; 63(3): 512-515. https://doi.org/10.1111/j.1365-2621.1998.tb15775.x
15. Brizhik L, Musumeci F, Scordino A, Triglia A. The soliton mechanism of the delayed luminescence of biological systems. Europhys. Lett. 2000; 52: 238-244. https://doi.org/10.1209/epl/i2000-00429-5

16. Brizhik L, Scordino A, Triglia A, Musumeci F. Delayed luminescence of biological systems arising from correlated many-soliton states. Phys. Rev. E 2001; 64(3): 031902. https://doi.org/10.1103/PhysRevE.64.031902

17. Brizhik L, Musumeci F, Scordino A, Tedesco M, Triglia A. Nonlinear dependence of the delayed luminescence yield on the intensity of irradiation in the framework of a correlated soliton mode. Phys. Rev. E 2003; 67(2): 021902. https://doi.org/10.1103/PhysRevE.67.021902

18. Scordino A, Grasso R, Gulino M, Lanzanò L, Musumeci F, Privitera G, et al. Delayed luminescence from collagen as arising from soliton and small polaron states. Int. J. Quantum Chem. 2010; 110(1): 221-229. https://doi.org/10.1002/qua.22010

19. Backhaus WG, Kliegl R, Werner JS. Color vision: Perspectives from different disciplines. Walter de Gruyter, Berlin; 2011.

20. Arias R, Lee TC, Logendra L, Janes H. Correlation of lycopene measured by HPLC with the L*, a*, b* color readings of a hydroponic tomato and the relationship of maturity with color and lycopene content. J. Agric. Food Chem. 2000; 48(5): 1697-1702. https://doi.org/10.1021/jf990974e

21. Pang J, Yang M, Fu J, Zhao X, van Wijk E, Wang M, et al. Classification of Chinese herbs based on the cluster analysis of delayed luminescence. Luminescence 2016; 31(2): 491–498. https://doi.org/10.1002/bio.2987.

22. Sun M, Li L, Wang M, van Wijk E, He M, van Wijk R, et al. Effects of growth altitude on chemical constituents and delayed luminescence properties in medicinal rhubarb. J. Photochem. Photobiol. B. 2016; 162: 24–33. https://doi.org/10.1016/j.jphotobiol.2016.06.018

23. Scordino A, Triglia A, Musumeci F, Grasso F, Rajfur Z. Influence of the presence of atrazine in water on the in-vivo delayed luminescence of *Acetabularia acetabulum*. J. Photochem. Photobiol. B. 1996; 32: 11–17. https://doi.org/10.1016/1011-1344(95)07213-6

24. Grasso R, Musumeci F, Triglia A, Brizhik L, Scordino A. Impact of structure on the delayed luminescence of d-Glucose-based polymer chains. J. Photochem. Photobiol. B. 2019; 198: 111589. https://doi.org/10.1016/j.jphotobiol.2019.111589

25. Austin RH, Beeson KW, Eisenstein L, Frauenfelder H, Gunsalus IC. Dynamics of ligand binding to myoglobin. Biochemistry 1975; 14(24): 5355-5373. https://doi.org/10.1021/bi00695a021

26. Frauenfelder H, Wolynes PG, Austin RH. Biological Physics. Rev. Mod. Phys. 1999; 71(2): S419-S430. https://doi.org/10.1103/RevModPhys.71.S419

27. Palazzo G, Mallardi A, Hochkoeppler A, Cordone L, Venturosi G. Electron transfer kinetics in photosynthetic reaction centers embedded in trehalose glasses: trapping of conformational substates at room temperature. Biophys. J. 2002; 82(2): 558-568. https://doi.org/10.1016/S0006-3495(02)75421-0

28. Popp FA, Yan Y. Delayed luminescence of biological systems in terms of coherent states. Phys. Lett. A 2002; 293(1-2): 93–97. https://doi.org/10.1016/S0375-9601(01)00831-3

29. Gormley R, Egan S. Firmness and colour of the fruit of some tomato cultivars from various sources during storage. J. Sci. Food Agric. 1978; 29(6): 534-538. https://doi.org/10.1002/jsfa.2740290607

30. Kaur D, Sharma R, Wani AA, Gill BS, Sogi DS. Physicochemical changes in seven tomato (*Lycopersicon esculentum*) cultivars during ripening. Int. J. Food Prop. 2006; 9(4): 747-757. https://doi.org/10.1080/10942910600575716.

31. Li N, Wang J, Wang B, Huang S, Hu J., Yang T, et al. Identification of the carbohydrate and organic acid metabolism genes responsible for Brix in tomato fruit by transcriptome and metabolome analysis. Front. Genet. 2021; 12: 714942. https://doi.org/10.3389/fgene.2021.714942

32. Magwaza LS, Opara UL Analytical methods for determination of sugars and sweetness of horticultural products - A review. Sci. Hortic. 2015; 184: 179-192. https://doi.org/10.1016/j.scienta.2015.01.001.

33. Wadood SA, Boli G, Xiaowen Z, Hussain I, Yimin W. Recent development in the application of analytical techniques for the traceability and authenticity of food of plant origin. Microchemical J. 2020; 152: 104295. https://doi.org/10.1016/j.microc.2019.104295

34. Fiamegos Y, Dumitrascu C, Papoci S, de la Calle MB. Authentication of PDO paprika powder (Pimentón de la Vera) by multivariate analysis of the elemental fingerprint determined by ED-XRF. A feasibility study. Food Control 2021; 120: 107496. https://doi.org/10.1016/j.foodcont.2020.107496

35. Ghidotti M, Fiamegos Y, Dumitrascu C, de la Calle MB. Use of elemental profiles to verify geographical origin and botanical variety of Spanish honeys with a protected denomination of origin. Food Chem. 2021; 342: 128350. https://doi.org/10.1016/j.foodchem.2020.128350

36. Martinez L, He L. Detection of mycotoxins in food using surface-enhanced Raman spectroscopy: A review. ACS Appl. Bio Mater. 2020; 4(1): 295-310. https://doi.org/10.1021/acsabm.0c01349

37. Neng J, Zhang Q, Sun P. Application of surface-enhanced Raman spectroscopy in fast detection of toxic and harmful substances in food. Biosens. Bioelectron. 2020; 167: 112480. https://doi.org/10.1016/j.bios.2020.112480

38. Kang J, Kim T, Jeong D, Lee H. Effect of dehydration stress on delayed luminescence in plant leaves. J. Korean Phys. Soc. 2008; 52:132–136. https://doi.org/10.3938/jkps.52.132.

39. Goltsev V, Zaharieva I, Chernev P, Strasser RJ. Delayed fluorescence in photosynthesis. Photosynth. Res. 2009; 101(2–3): 217–232. https://doi.org/10.1007/s11120-009-9451-1

40. Sun M., Chang W.T., van Wijk E., He M., van Wijk R., Wang M. Application of delayed luminescence method on measuring of the processing of Chinese herbal materials. Chinese Med. 2018; 13(1): 43. https://doi.org/10.1186/s13020-018-0202-0

41. Sun M, Wang S, Jing Y, Li L, He M, Jia Y, et al. Application of delayed luminescence measurements for the identification of herbal materials: a step toward rapid quality control. Chinese Med. 2019; 14(1): 47. https://doi.org/10.1186/s13020-019-0269-2

42. Stolz P, Wohlers J, Mende G. Measuring delayed luminescence by FES to evaluate special quality aspects of food samples–an overview. Open Agric. 2019; 4(1): 410-417. https://doi.org/10.1515/opag-2019-0039

43. Kader AA, Stevens MA, Albright-Holton M, Morris LL, Agazi M Effect of fruit ripeness when picked on flavor and composition in fresh market tomatoes. J. Am. Soc. Hortic. Sci. 1977; 102(6): 724-731 https://doi.org/10.21273/JASHS.102.6.724.

44. Chen W, Xing D, Chen W. Rapid detection of *Aspergillus flavus* contamination in peanut with novel delayed luminescence spectra. Photochem. Photobiol. 2005; 81(6): 1361-1365. https://doi.org/10.1562/2005-05-23-RC-538

Figure

Click here to access/download;Figure;Fig1.tif

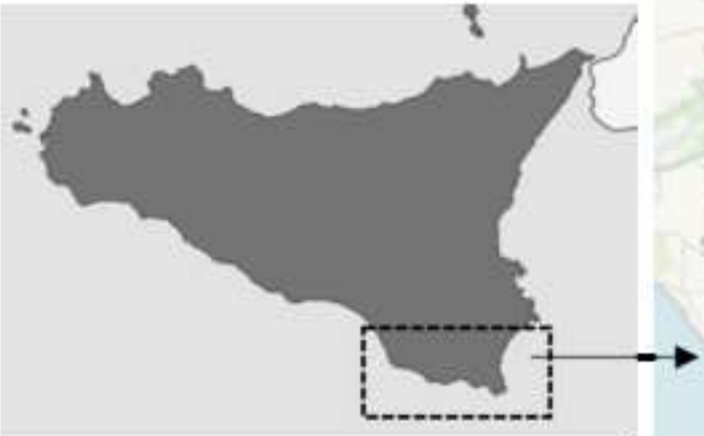

Area of origin	Sample ID
Marina di Ragusa	Lot 1
Santa Croce Camerina	Lot 2
Ispica	Lot 3
Ispica	Lot 4

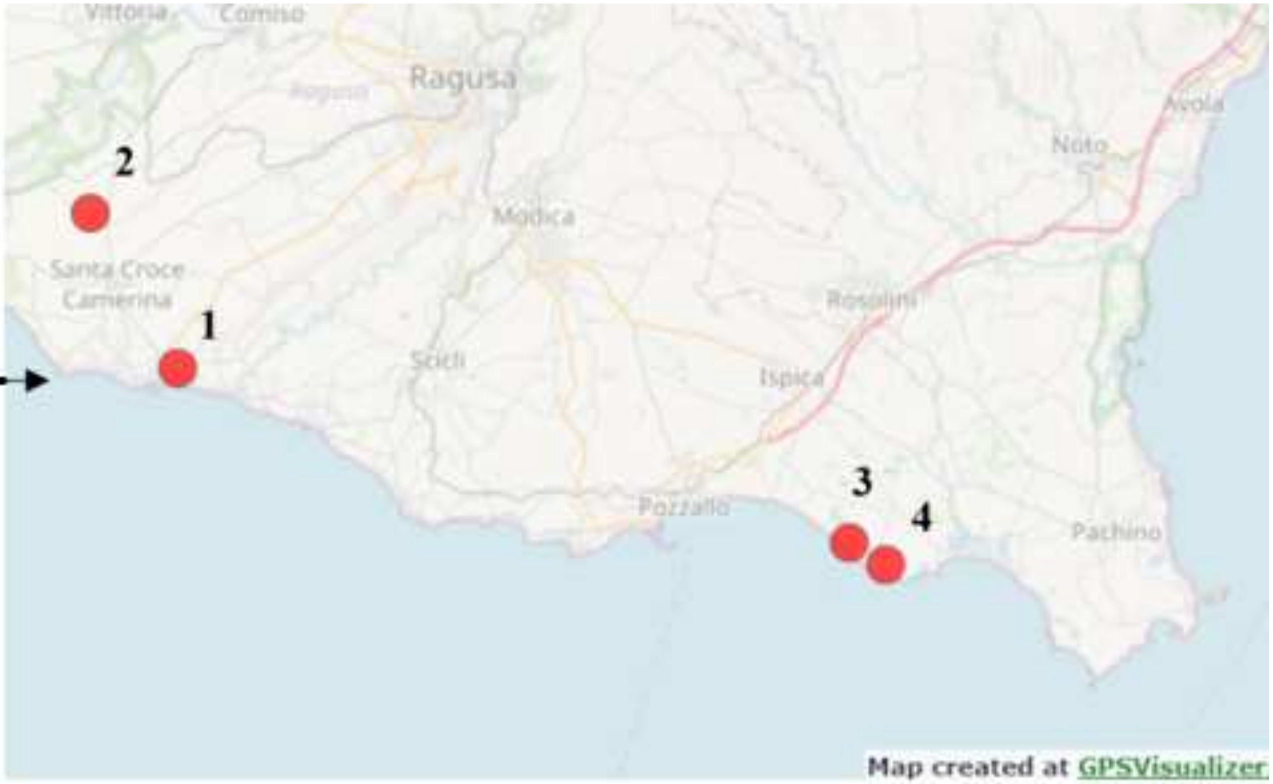

Figure

Click here to access/download;Figure;Fig2.tif

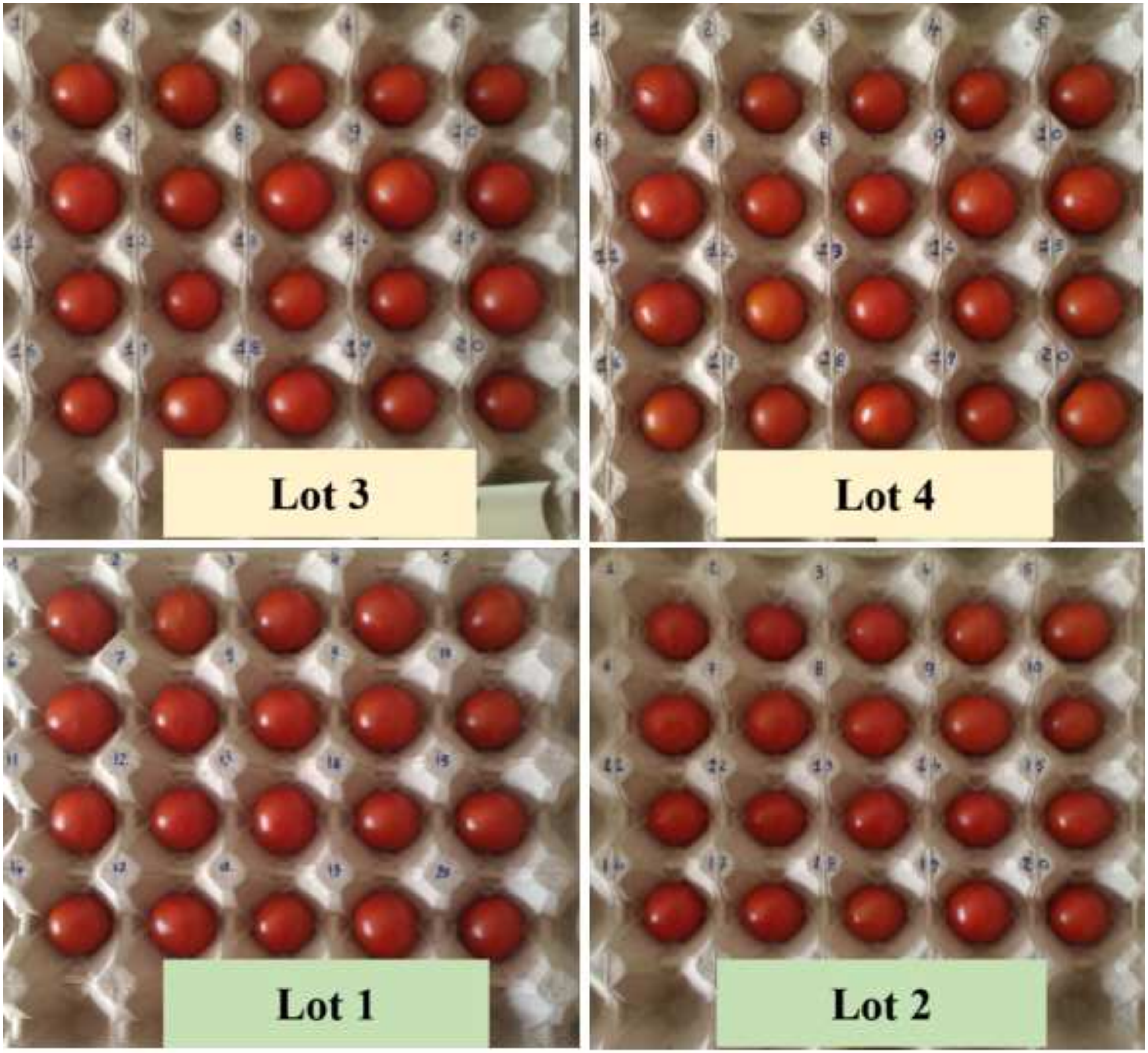

Figure

Click here to access/download;Figure;Fig3.tif

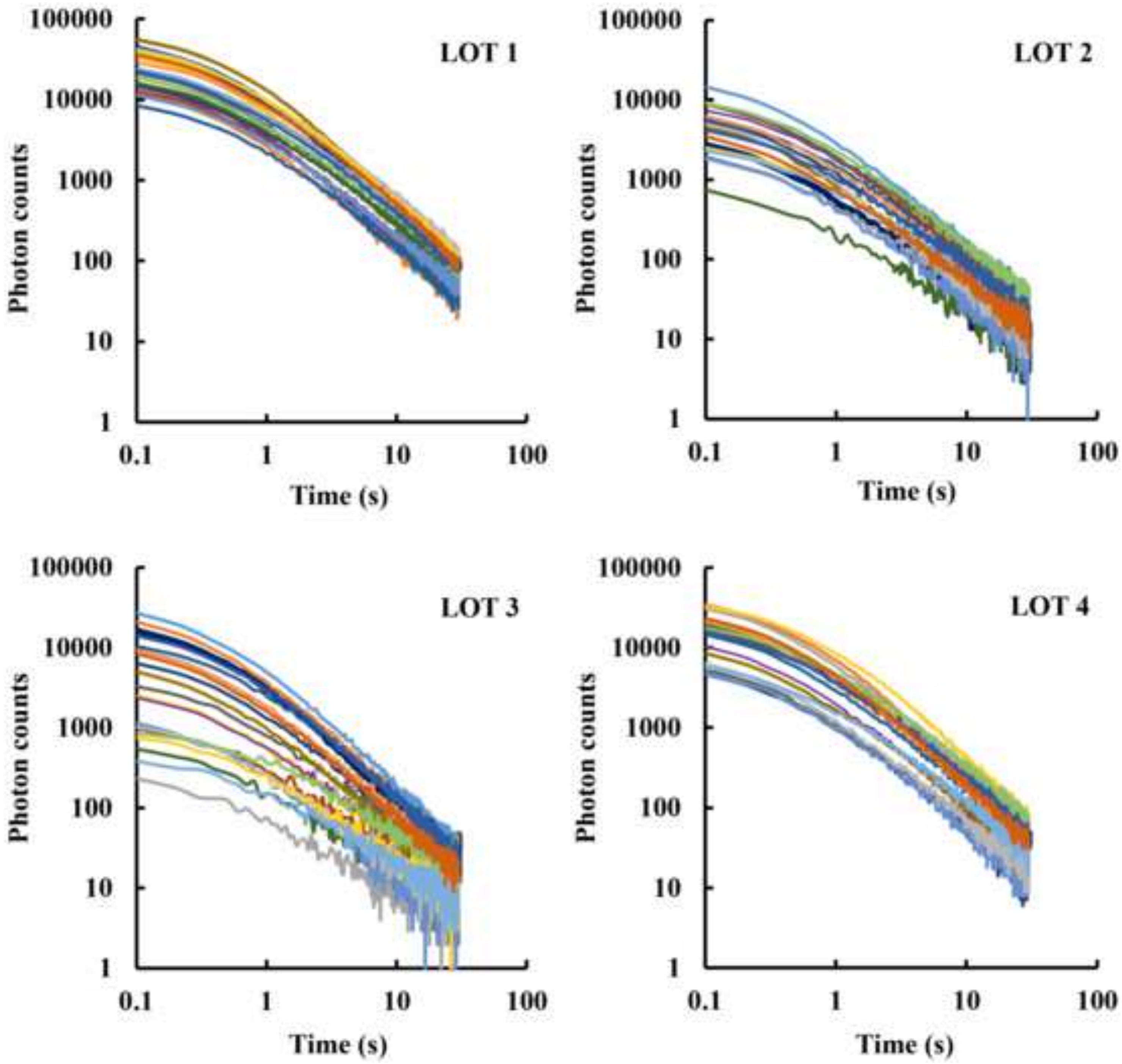

Click here to access/download;Figure;Fig4.tif

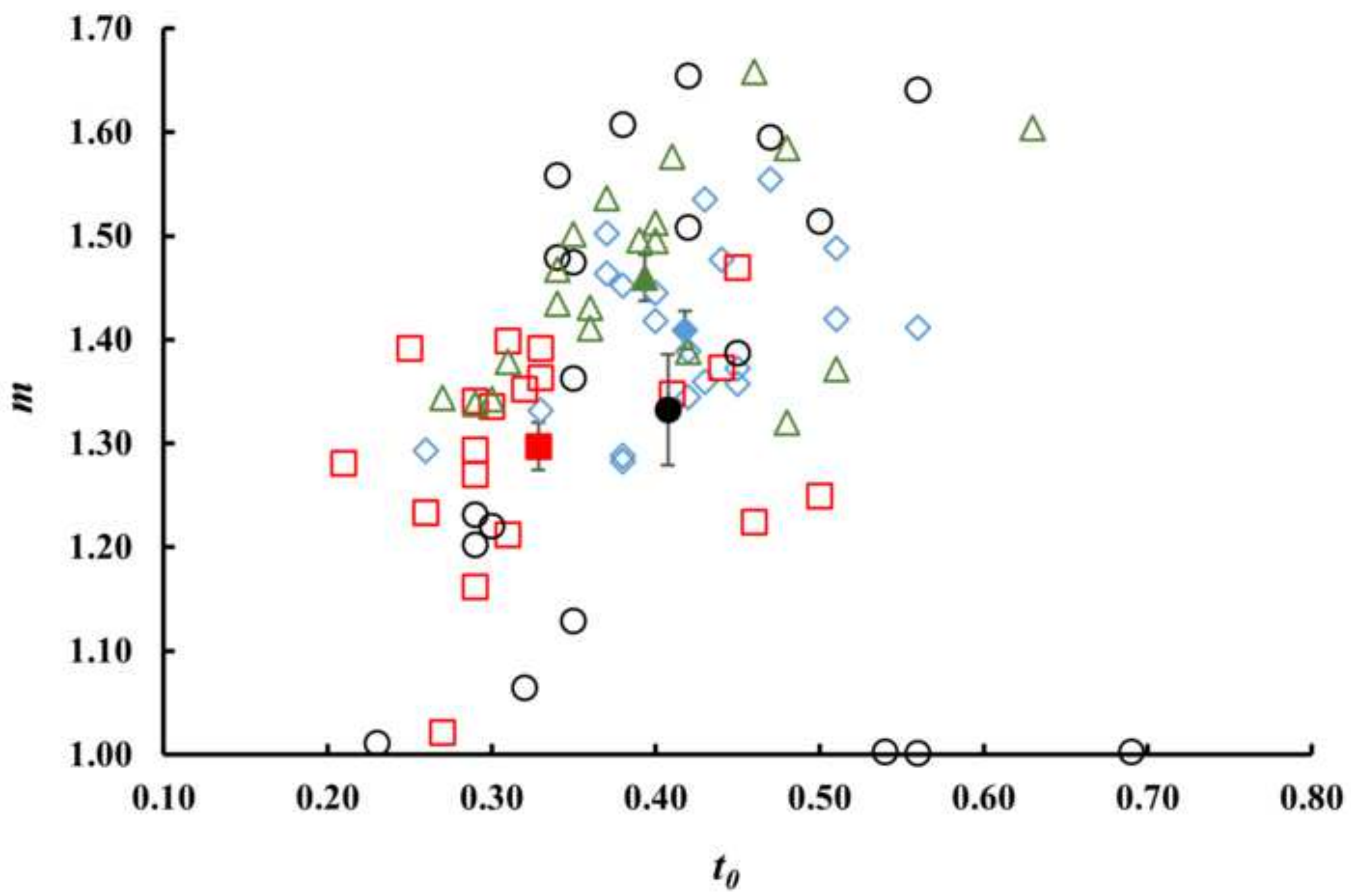

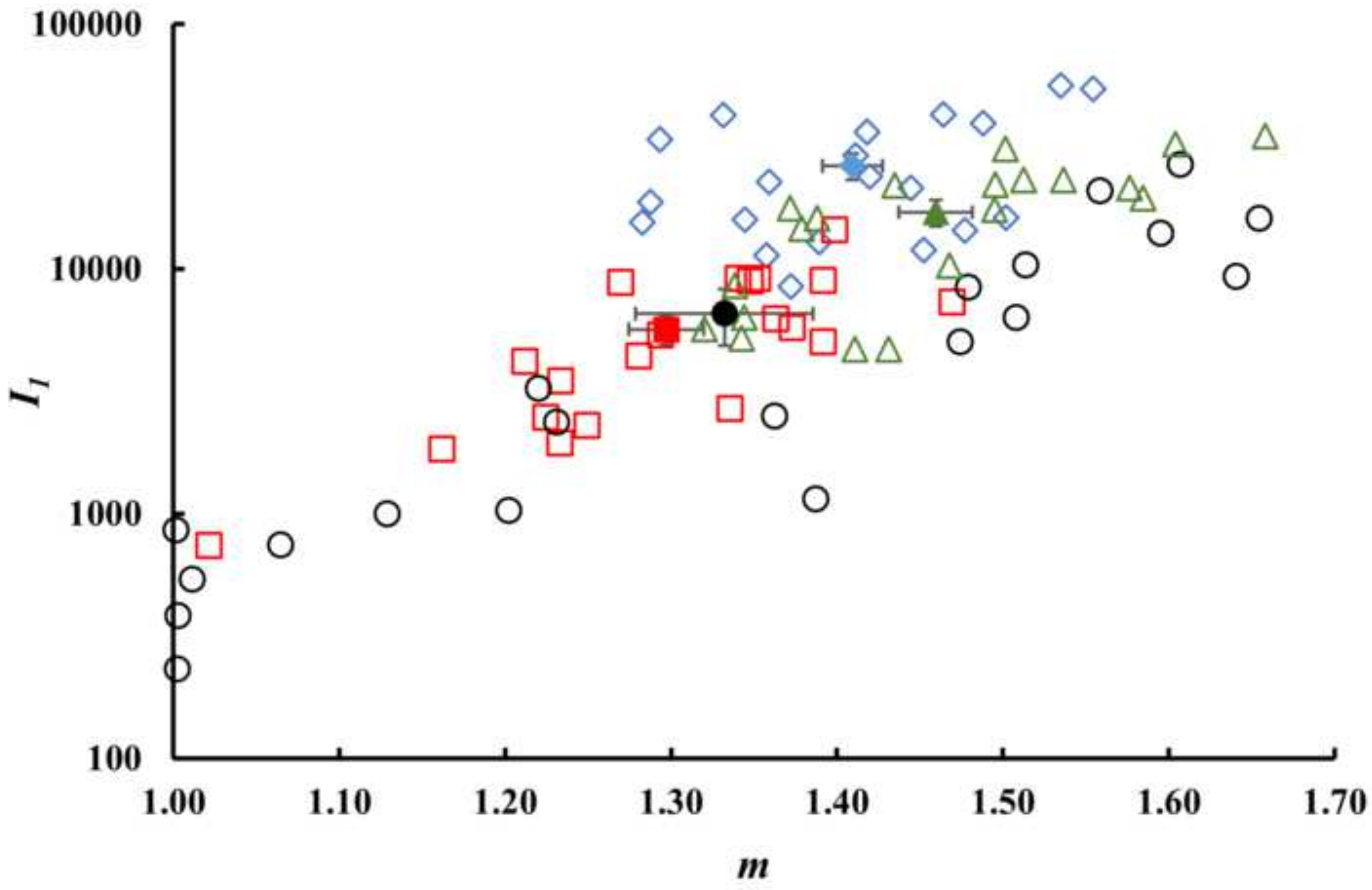
100000
10000
1000
100
I_1
1.00
1.10
1.20
1.30
1.40
1.50
1.60
1.70
m

Figure Click here to access/download;Figure;Fig6.tif

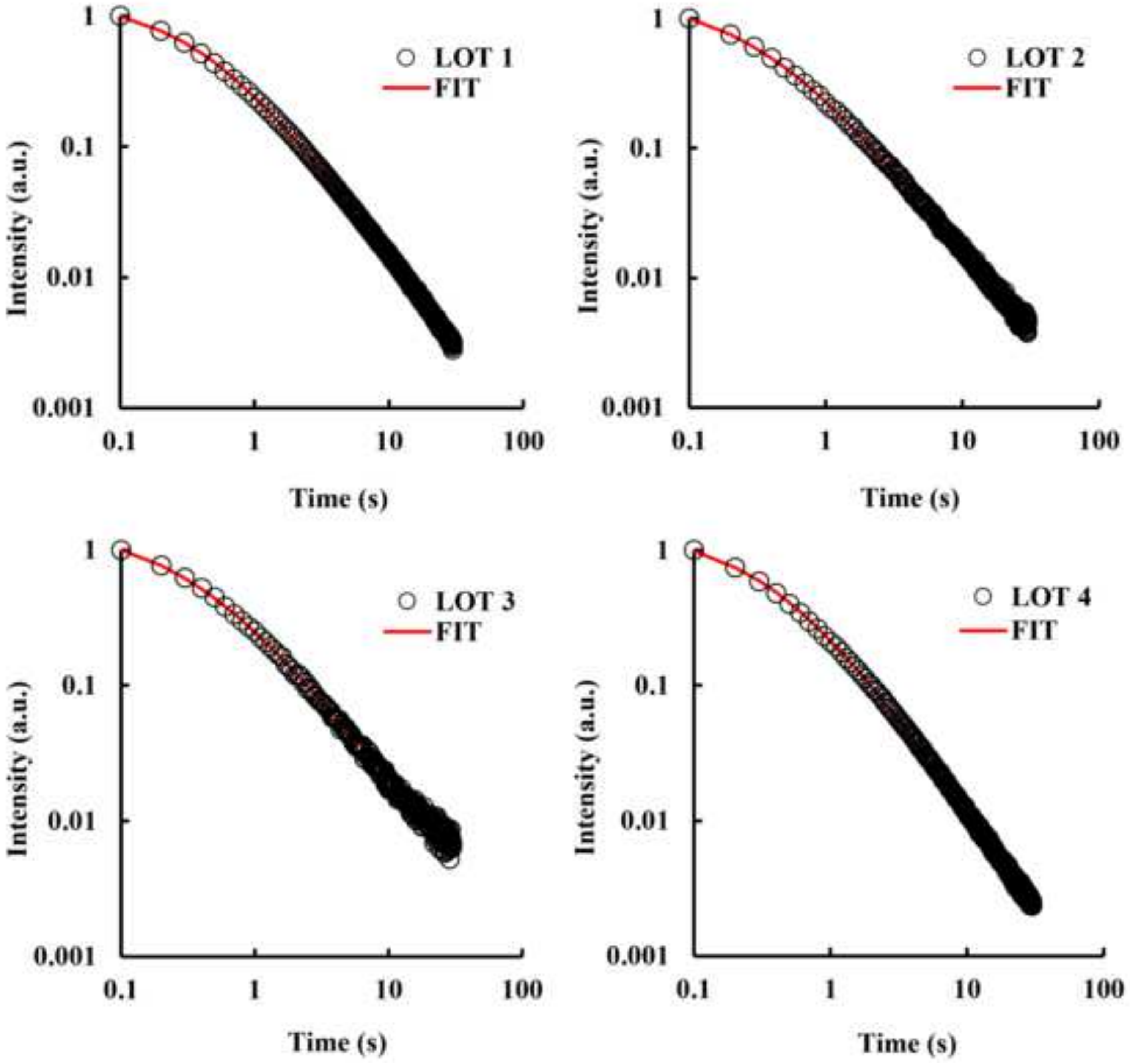

Figure

Click here to access/download;Figure;Fig7.tif

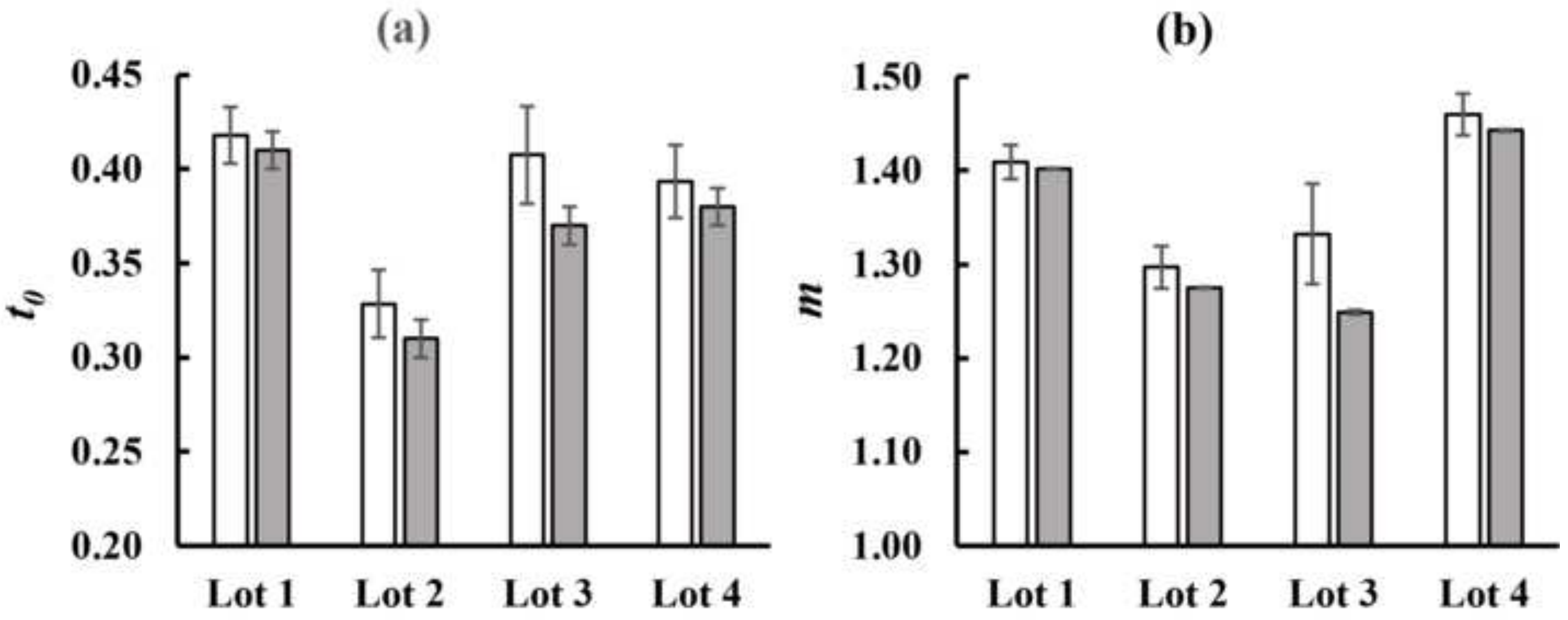

 Click here to access/download;Figure;Fig8.tif

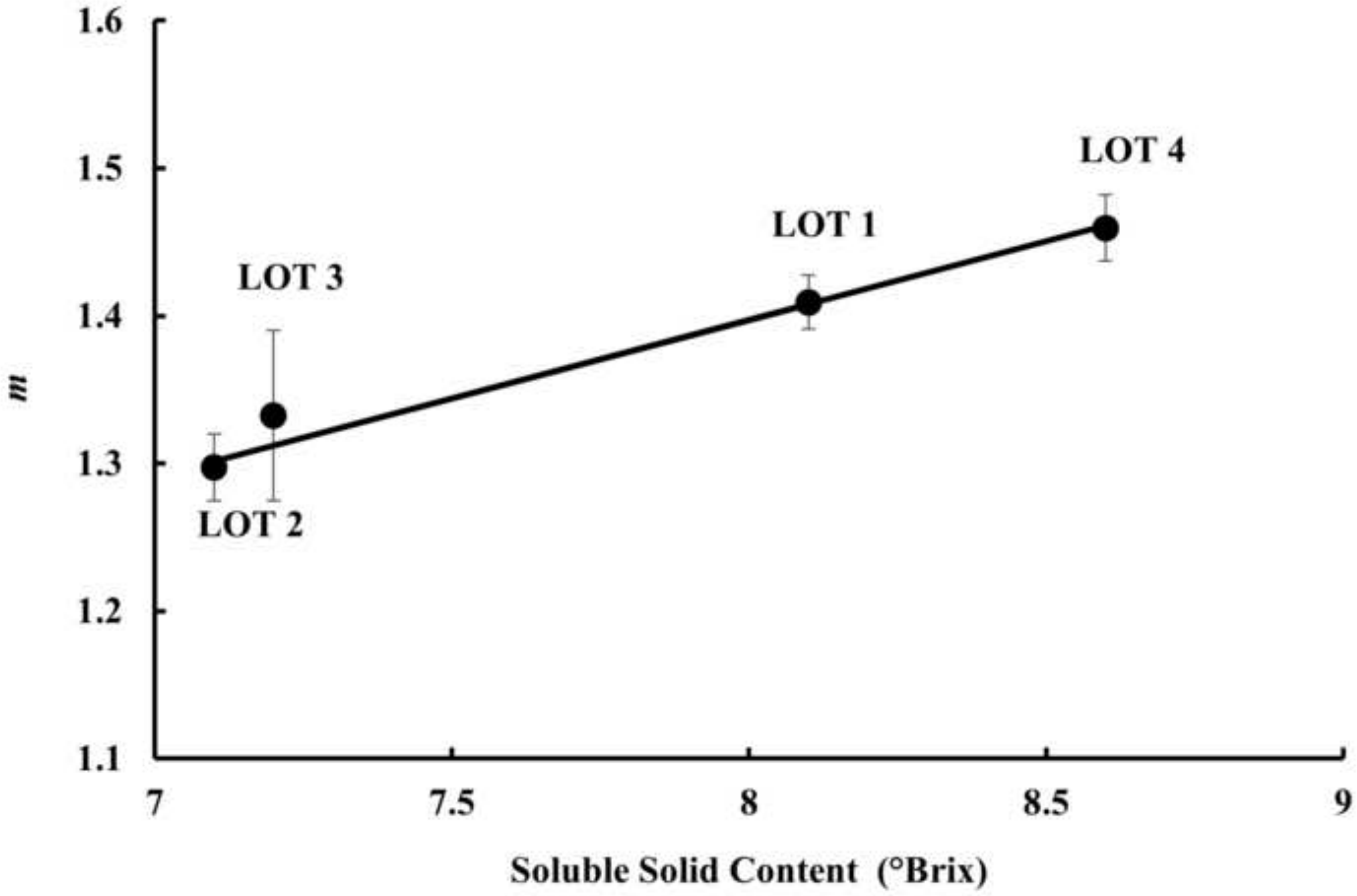

Supplement: Attachment — Submitted filename: PONE-D-23-08412_reviewer_edited.pdf [file pone.0286383.s004.pdf]
