# Supplementary material for: Applications of Delayed Luminescence for tomato fruit quality assessment across varied Sicilian cultivation zones
Source: PLoS One. 2023 Jun 1;18(6):e0286383. doi: 10.1371/journal.pone.0286383 (PMC10234554; doi:10.1371/journal.pone.0286383)
Supplement: Attachment — Submitted filename: Response to Reviewers.docx [file pone.0286383.s005.docx]

Catania, May 12, 2023

POINT BY POINT ANSWERS TO THE REVIEW TEAM

**Ms. Ref. No.: PONE-D-23-08412**

Dear Editor,

We thank the Staff Editors and the Reviewers for the useful comments and suggestions. Please, find in the following the details of our replies and modifications according to the Referee comments and the Journal Requirements (reported in blue):

**Referee comments**

Reviewer #1:

Q1) Line 118 should be physicochemical.

A1) According to the suggestion, “physic-chemical” has been replaced by “physicochemical” in the line 118 of the manuscript and in various other parts of text.

Q2) If statistical letters are written as superscripts will be much easy to understand.

A2) Concerning this suggestion, we wrote the statistical letters as superscripts, both in Table 1 and in Table 2.

Q3) In the paper says it is difficult to apply DL for all produced tomatoes at least on a farm. So how can the method be applicable?

A3) Actually, we do not understand this question. Nowhere in the paper does it say “it is difficult to apply DL for all produced tomatoes at least on a farm”, neither such a conclusion can be drawn by our experimental study. Conversely, our experimental study supports the idea that, after standardization, a no-destructive method based on Delayed Luminescence to check single fruit quality could be implemented.

Reviewer #2:

Generally well written, however, some revisions have been suggested. Please see attachments.

A) We have revised the manuscript, according to the comments suggested in attachments. As requested, we have also rewritten some sentences to clarify our concepts in lines 79-84, 87-91, 230-236, 371-375, 385-389 and 403-408 of the manuscript.

**Journal Requirements**

R1) Please ensure that your manuscript meets PLOS ONE's style requirements, including those for file naming.

A1) Thank you for your suggestion. We checked again the style of our manuscript, aligning it with that required by PLOS ONE journal.

R2) In your Methods section, please provide additional information regarding the permits you obtained for the work. Please ensure you have included the full name of the authority that approved the field site access and, if no permits were required, a brief statement explaining why.

A2) According to this requirement, we have provided additional information regarding the permits obtained for the work, reporting in the “Materials and methods” section the sentence “All Lots were kindly provided by farm owners” in lines 104-105 of the manuscript. In addition, we have included the Acknowledgments section to provide further clarifications on the supply of tomato samples.

R3) We note that the grant information you provided in the ‘Funding Information’ and ‘Financial Disclosure’ sections do not match. When you resubmit, please ensure that you provide the correct grant numbers for the awards you received for your study in the ‘Funding Information’ section.

A3) Regarding this requirement, we have rewritten the information regarding the received fundings, according to journal rules reported in the “Financial Disclosure Statement”. We have written the full name of research projects, which uniquely identify the grants. Please, look at the new sentence below:

Financial support was provided from the University of Catania, Italy, in the framework of the Piano di Incentivi per la Ricerca di Ateneo (Pia.Ce.Ri.) 2020-2022, Linea 2. GC and AS received the funding for the following research projects: “Investigation of phytopathological problems of the main Sicilian productive contexts and eco-sustainable defense strategies (MEDIT-ECO)” (GC) and “Sviluppo di Nanomateriali e Tecnologie Innovative per Smart detection” (NaTI4Smart) (AS). This research was also funded by the Ministry of University and Research, Italy, Area di Specializzazione Agrifood, Research Project “POFACS - Shelf-life, quality and safety of high-convenience fruit and vegetables” (ARS01-00640) (GS). The funders had no role in study design, data collection and analysis, decision to publish, or preparation of the manuscript.

R4) Thank you for stating the following financial disclosure:

"Funded by Incentive Plan for Research (PIA.CE.RI.) 2020-22 line 2 of the University of Catania, Research Projects “NaTI4Smart” and “MEDIT ECO”, National Operational Programme (PON) on Research and Innovation 2014-2020 “POFACS (ARS01_00640)” - Italian Ministry of University and Research. The funders had no role in study design, data collection and analysis, decision to publish, or preparation of the manuscript.

We note that one or more of the authors is affiliated with the funding organization, indicating the funder may have had some role in the design, data collection, analysis or preparation of your manuscript for publication; in other words, the funder played an indirect role through the participation of the co-authors. If the funding organization did not play a role in the study design, data collection and analysis, decision to publish, or preparation of the manuscript and only provided financial support in the form of authors' salaries and/or research materials, please do the following:

(1) Review your statements relating to the author contributions and ensure you have specifically and accurately indicated the role(s) that these authors had in your study. These amendments should be made in the online form.

(2) Confirm in your cover letter that you agree with the following statement, and we will change the online submission form on your behalf:

“The funder provided support in the form of salaries for authors [insert relevant initials] but did not have any additional role in the study design, data collection and analysis, decision to publish, or preparation of the manuscript. The specific roles of these authors are articulated in the ‘author contributions’ section.

A4) Regarding this requirement, we have included the initials of the authors who received the funding, as you can see in the answer n.3. The funded projects did not provide support in the form of salaries for authors and the funders had no role in the study design, data collection and analysis, decision to publish, or preparation of the manuscript. These roles have been filled by the authors, as we have reported in the ‘author contributions’ section”. Thus, we agree that you change the online submission form on our behalf only for the sentence reported in the answer n. 3.

R5) In your Data Availability statement, you have not specified where the minimal data set underlying the results described in your manuscript can be found.

PLOS defines a study's minimal data set as the underlying data used to reach the conclusions drawn in the manuscript and any additional data required to replicate the reported study findings in their entirety. All PLOS journals require that the minimal data set be made fully available. For more information about our data policy, please see: <http://journals.plos.org/plosone/s/data-availability>.

Upon re-submitting your revised manuscript, please upload your study’s minimal underlying data set as either Supporting Information files or to a stable, public repository and include the relevant URLs, DOIs, or accession numbers within your revised cover letter. For a list of acceptable repositories, please see <http://journals.plos.org/plosone/s/data-availability#loc-recommended-repositories>

Any potentially identifying patient information must be fully anonymized.

Important: If there are ethical or legal restrictions to sharing your data publicly, please explain these restrictions in detail. Please see our guidelines for more information on what we consider unacceptable restrictions to publicly sharing data: <http://journals.plos.org/plosone/s/data-availability#loc-unacceptable-data-access-restrictions>. Note that it is not acceptable for the authors to be the sole named individuals responsible for ensuring data access. We will update your Data Availability statement to reflect the information you provide in your cover letter.

A5) We accept that all data underlying our results are fully available without restriction. Therefore, we agree to facilitate access to data. For this reason, as you suggest, we have included the “Supplementing Information” section within the manuscript. In detail, we have prepared and submitted a datasheet as Excel file, containing DL datasets for all Lots under study (photon number released by fruits in a time interval of 30 s after excitation) and two Supplementary Figures, which clarify results described in lines 371-373 of the manuscript. We cited these supporting data into the manuscript text in the line 225 using the item “S1 Appendix” and in the line 373 using the items “S1 and S2 Figs”.

R6) Please amend either the title on the online submission form (via Edit Submission) or the title in the manuscript so that they are identical.

A6) The title has been changed according to the suggestion of Reviewer 2. As required, we have also modified the title on the online submission form (Edit submission), so that it is identical to the new title in the manuscript.

R7) We note that Figure 1 in your submission contain map images which may be copyrighted.

All PLOS content is published under the Creative Commons Attribution License (CC BY 4.0), which means that the manuscript, images, and Supporting Information files will be freely available online, and any third party is permitted to access, download, copy, distribute, and use these materials in any way, even commercially, with proper attribution. For these reasons, we cannot publish previously copyrighted maps or satellite images created using proprietary data, such as Google software (Google Maps, Street View, and Earth). For more information, see our copyright guidelines:

<http://journals.plos.org/plosone/s/licenses-and-copyright>.

…………………………….……………………………………

If you are unable to obtain permission from the original copyright holder to publish these figures under the CC BY 4.0 license or if the copyright holder’s requirements are incompatible with the CC BY 4.0 license, please either i) remove the figure or ii) supply a replacement figure that complies with the CC BY 4.0 license. Please check copyright information on all replacement figures and update the figure caption with source information. If applicable, please specify in the figure caption text when a figure is similar but not identical to the original image and is therefore for illustrative purposes only.

A7) Concerning the Figure 1, which contain map images which could be copyrighted, we have supplied a replacement figure that complies with the CC BY 4.0 license. The new maps were freely downloaded from NASA Earth Observatory (<http://earthobservatory.nasa.gov/>) and Earth Resources Observatory and Science – EROS – Center ([http://eros.usgs.gov/#](http://eros.usgs.gov/)), as suggested by the Journal PLOS ONE. We have updated the figure caption, indicating source information. In detail, for the caption of the Fig. 1, we report this sentence:

“Fig 1. Area of origin of tomato Lots collected for this study. Lots 3 and 4 were certified with the label Protected Geographical Indication (PGI). The imageries were freely downloaded from NASA Earth Observatory (http://earthobservatory.nasa.gov/) and USGS EROS (Earth Resources Observatory and Science – EROS – Center, http://eros.usgs.gov/#). The figure on the right side is similar but not identical to the original image and is therefore for illustrative purposes only.”

R8) Please review your reference list to ensure that it is complete and correct. If you have cited papers that have been retracted, please include the rationale for doing so in the manuscript text or remove these references and replace them with relevant current references. Any changes to the reference list should be mentioned in the rebuttal letter that accompanies your revised manuscript. If you need to cite a retracted article, indicate the article’s retracted status in the References list and also include a citation and full reference for the retraction notice.

A8) Regarding this requirement, no cited paper has been retracted. Some changes have been performed on reference list and, in particular, on references n. 2, 5, 11 and 19. In detail, we added some hyperlinks (references n. 2 and 11), added or fixed some mistakes regarding DOI number (references n. 2, 5 and 11) and reported the ISBN number for book cited in line 485 of the manuscript (reference n. 19).

Yours Sincerely, Salvina Panebianco

PhD, post-doctoral research at Di3A

University of Catania
